# Population Response Propagation to Extrastriate Areas Evoked by Intracortical Electrical Stimulation in V1

**DOI:** 10.3389/fncir.2016.00006

**Published:** 2016-02-12

**Authors:** Tamás D. Fehérvári, Tetsuya Yagi

**Affiliations:** Bio-System and Device Laboratory, Division of Electrical, Electronic and Information Engineering, Graduate School of Engineering, Osaka UniversityOsaka, Japan

**Keywords:** mouse, visual cortex, voltage-sensitive dye, intracortical electrical stimulation, *in vivo*, interareal transmission, neural circuits

## Abstract

The mouse visual system has multiple extrastriate areas surrounding V1 each with a distinct representation of the visual field and unique functional and connectivity profiles, which are believed to form two parallel processing streams, similar to the ventral and dorsal streams in primates. At the same time, mouse visual areas have a high degree of interconnectivity, in particular V1 sends input to all higher visual areas. The study of these direct connections can further our understanding of the cortical processing of visual signals in the early mammalian cortex. Several studies have been published about the anatomy of these connections, but an *in vivo* electrophysiological characterization and comparison of the transmission to multiple extrastriate areas has not yet been reported. We used intracortical electrical stimulation combined with RH1691 VSD imaging in adult C57BL/6 mice in urethane anesthesia to analyze interareal transmission from V1 to extrastriate areas in superficial cortical layers. We found seven extrastriate response sites (five lateral, two medial) in a spatial pattern similar to area maps of the mouse visual cortex and, by shifting the location of V1 stimulation, demonstrated that the evoked responses in LM and AL were in accordance with the visuotopic mappings of these areas known from anatomy and *in vivo* studies. These two sites, considered to be gateways to their processing streams, had shorter latencies and faster transmission speeds than other extrastriate response sites. Short latency differences between response sites, and that TTX injection into LM reduced but did not eliminate other extrastriate responses indicated that the evoked cortical activity was, at least partially, transmitted directly from V1 to extrastriate areas. This study reports on analysis of interareal transmission from V1 to multiple extrastriate areas in mouse using intracortical electrical stimulation *in vivo*.

## Introduction

The mouse primary visual cortex (V1) is surrounded by multiple extrastriate areas, each containing a distinct representation of the visual field and displaying a unique selectivity to spatiotemporal features of visual stimuli and cortico-cortical connectivity profiles (Wagor et al., 1980; Wang and Burkhalter, 2007, 2013; Andermann et al., 2011; Marshel et al., 2011; Wang et al., 2011, 2012; Roth et al., 2012). It is believed that mouse visual areas form two parallel processing streams, which are possibly analogous to the ventral and dorsal streams in primates (Marshel et al., 2011; Wang et al., 2011, 2012; Wang and Burkhalter, 2013). However, there is also a high degree of interconnectivity between mouse visual areas. In particular, unlike in primates, V1 provides input to essentially all extrastriate visual areas (Olavarria et al., 1982; Olavarria and Montero, 1989; Wang and Burkhalter, 2007).

The dynamics of V1-extrastriate interareal connections have been analyzed in mouse visual cortex slice studies using intracortical electrical stimulation (Dong et al., 2004), and photostimulation (De Pasquale and Sherman, 2011; Yang et al., 2013). Stimulation in V1 has the advantage that the evoked cortical activity originates locally. In contrast, visual stimuli are processed in several stages prior to the visual cortex (retina, superior colliculus, and thalamus), some of which also send input directly to higher visual areas (Caviness and Frost, 1980; Simmons et al., 1982; Tohmi et al., 2014), which makes the investigation of direct V1-extrastriate transmission difficult. These slice studies focused only on connections between V1 and LM, however, and a simultaneous study of transmission to multiple extrastriate areas has not yet been reported in mouse.

In this work, we used intracortical electrical stimulation to characterize V1-extrastriate connections *in vivo*. We used single-pulse current stimuli in V1 layer II/III and voltage-sensitive dye (VSD) imaging in adult mice under urethane anesthesia, and recorded high resolution (100 × 100 pixel) images of mesoscopic-scale cortical activity in superficial cortical layers in a wide field of view (approximately 3 × 3 mm^2^) that allowed us to simultaneously observe cortical activity in multiple higher visual areas. This experimental setup was similar to that in the pioneering study of Orbach and Van Essen (1993). Aside from a different animal model, their detector had a limited field of view that could only record from one visual area at one time. Our goal was to analyze the spatiotemporal dynamics of V1-extrastriate transmission, and by comparing evoked activity in multiple extrastriate areas to find out if differences between those areas could be revealed with this stimulation method.

## Materials and methods

The same experimental methodology was followed as reported in Fehérvári et al. (2015) with small modifications.

### Ethics statement

All experiments were approved by the Institutional Animal Care and Use Committee of the Graduate School of Engineering, Osaka University (permit number 17-6-0), and were conducted in accordance with the Guiding Principles for the Care and Use of Animals in the Field of Physiological Sciences of the Physiological Society of Japan and Guidelines for Animal Experiments of Osaka University. All surgery and recording was performed under urethane anesthesia, and all efforts were made to minimize suffering. The level of anesthesia was assessed by pinching and additional small doses of urethane were injected when necessary. The animals were euthanized by decapitation after administration of an overdose of the anesthetic.

### Animals

Experiments were performed on 37 adult C57BL/6 mice (8–20 weeks; CLEA Japan, Inc., Tokyo, Japan), kept in a room under a 12-h light/dark cycle and provided food and water *ad libitum*. Four of the mice were used for experiments with tetrodotoxin (TTX), other results are based on the remaining 33 animals.

### Surgery and VSD staining

Anesthesia was induced with intraperitoneal (i.p.) injection of urethane (1.25 g/kg body weight) and further small doses were added when necessary to maintain level of anesthesia. Atropine (0.01 mg, i.p.) and dexamethasone (0.02 mg, i.p.) were administered to suppress mucus secretion and brain oedema, respectively. The skin was shaved and treated with a local anesthetic (Xylocaine 20 mg/mL, AstraZeneca) before making incisions. Tracheotomy and cannulation were performed to prevent blockage of the upper airways, and a gentle flow of oxygen was directed at the opening of the inserted tube to reduce the risk of hypoxia. The animal was placed in a stereotaxic apparatus (SR-15; Narishige, Tokyo, Japan). Rectal temperature was maintained at 36.8°C with a heating pad. Craniotomy was performed on the right posterior parietal bone to expose the visual cortex (0.5–4 mm from the midline and 0–3.5 mm from the right lambdoid suture). The dura was left intact. To create a chamber above the exposed cortex, an ~1-mm length silicone-rubber tube (inner diameter, 6.5 mm) was attached to the right posterior parietal bone using dental resin. Bleeding from the dura was stopped completely and the dura was dried thoroughly to increase its permeability (Xu et al., 2007). Staining was performed by bath application of RH1691 (Optical Imaging, Rehovot, Israel; 1 mg/mL in saline with 1.96 U/mL heparin and 0.125% dimethyl sulfoxide) for 90 min and then rinsed multiple times and washed for more than 20 min with artificial cerebrospinal fluid (ACSF; 125 mM NaCl, 2.5 mM KCl, 0.9 mM NaH2PO4, 5 mM Na2HPO4, 1.2 mM CaCl2, 1.0 mM MgCl2, 2.5 mM D-glucose). During recordings, the exposed cortex was covered with 50–60 μL ACSF, which was replaced every 20–30 min.

### Intracortical stimulation

Glass microelectrodes filled with ACSF were inserted within a region of 0.5–1 mm anterior from the lambdoid suture and 2.5–3.5 mm lateral from the midline in order to ensure that the electrical stimulation was applied to V1 (Dräger, 1975; Fehérvári et al., 2015). The electrodes were bent in an L shape close to the tip. The shaft of the electrode was held horizontally by a micromanipulator (NMN-21; Narishige), enabling movement of the electrode in the confined space between the head of the animal and the object lens and minimizing obstruction of the imaging area, at the same time allowing near-perpendicular insertion of the tip into the cortex, which helped with the targeting of the stimulation site and depth. The electrodes had a tip diameter of 5–6 μm, ~1 MΩ resistance, and the tip was positioned at a depth of 250 μm below the dura (layer II/III), by first lowering it beyond and then retracting it to the target depth to release possible compression of brain tissue. Anodic-first biphasic current pulses (intensity 50 μA, phase duration 200 μs, no interphase interval) were delivered with an isolated current generator (STG2008; Multi Channel Systems, Reutlingen, Germany). All stimulation sites were at least 500 μm away from the estimated V1-extrastriatum borders (Fehérvári et al., 2015) (in medial, lateral, and anterior directions) to minimize interference with extrastriate response sites.

### Tetrodotoxin electrophoresis

Glass micropipettes identical to the ones used for stimulation were filled with 25 μM tetrodotoxin (TTX, Wako Pure Chemical Industries Ltd.) in saline. The ejection current was 5 μA applied for 5 min, 2–3 times. The electrode was under a constant 1 μA retaining current in between injection rounds to prevent leakage. Recovery data was recorded 1–2 h after the last application of TTX.

### Optical imaging and data processing

A fluorescence microscope (THT-microscope; Brainvision, Tokyo, Japan) was set onto the VSD-stained visual cortex, which was illuminated with an excitation light (central wavelength 630 nm). The emitted fluorescence signals, passing through a dichroic mirror, and a 665-nm long-pass filter, were collected by a CMOS-based imaging system (100 × 100 pixels; MiCAM-Ultima; Brainvision) for 256–1024 ms at a frame rate of 1 kHz. Acquisition was triggered by the R component of the ECG which made the reduction of vascular pulsation artifacts by subtraction possible. The digitalized data of the fluorescent images were analyzed in Matlab (The MathWorks, Natick, MA, USA) with custom-written procedures. Fluorescence values were expressed as a percentage change (ΔF/F) relative to the baseline (mean of fluorescence signal measured for 30 ms before stimulation). Recordings with and without stimulation were taken alternatingly at ~15 s intervals, and the average of the data without stimulation was subtracted from the one with stimulation. For each trial, stimulation was repeated 10–24 times and these recordings were averaged in order to reduce noise. The data was filtered with a 3 × 3 pixel^2^ Gaussian spatial averaging filter for the localization of response sites; a 5 × 5 pixel^2^ Gaussian spatial filter was used before latency measurement and for time courses shown in figures. A 3-frames-wide temporal averaging filter was used in video illustrations and image sequences only. No additional filtering was applied to the data.

### Latency measurement

Latencies were measured at thresholds defined as a percentage of the maximum peak amplitude at each pixel. Latency measured at *x*% of peak maximum is also referred to as *x*%-latency, e.g., 15%-latency. The crossing point of the time course and the threshold was calculated with linear interpolation between the two data points above and below the threshold.

### Latency thresholds for response identification, localization, and measurement

In all trials, independent responses were identified as local latency minima appearing on at least two latency maps with thresholds ≥10% apart, between level of statistical significance (3 × SD of baseline) and 80% of local peak maximum. The position of these independent responses was determined on the 50%-latency map as the center of the active region within up to 1 ms of the lowest latency (earliest appearance). Site latency and speed results were measured at 15% of peak maximum at such positions. The consideration behind these chosen thresholds was to preserve comparability between trials, and that 50%-latency offered a good signal-to-noise ratio for determining locations, whereas 15% provided information about early activation while still being above the level of statistical significance at most response sites.

### Statistical tests

Data are expressed as mean ± standard deviation (SD). Statistical significance was assessed by using Student's two-sample *t*-test for comparison of two population distributions and One-way analysis of variance (ANOVA) for multiple sample populations with the Tukey-Kramer honestly significant difference criterion for multiple comparison tests.

## Results

### V1 stimulation effects

**Figure 1:**
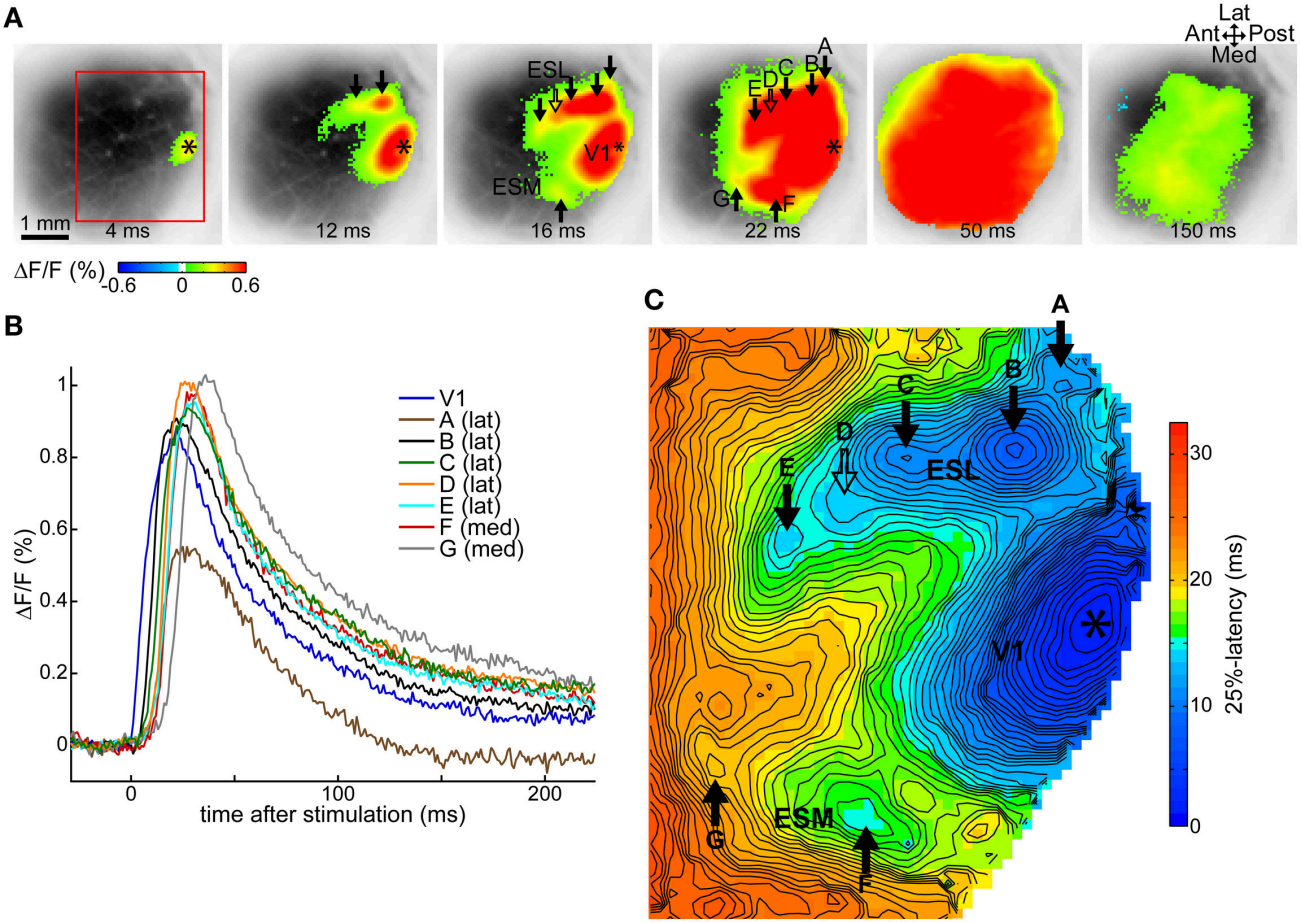
**Cortical activity following V1 stimulation**. Data from one trial, average of 12 repeated stimulations. **(A)** VSD signals following a 50 μA single-pulse current stimulus in V1 show statistically significant (above 3 × SD of pre-stimulus baseline) evoked activity around the stimulation site and separate secondary extrastriate spots. Frames taken at indicated delays after stimulation. **(B)** Time courses of the VSD fluorescence signal at labeled response locations. **(C)** Latency map at 25% of peak maximum at each pixel in the ROI demarcated with a red rectangle on **(A)**. **(A,C)** Asterisk, stimulation site; ESL, ESM, lateral and medial extrastriate region, respectively; solid arrow, independent responses (labels A–G except D); hollow arrow, response suggested by contour protrusion, not independent here (label D); Ant, anterior; Post, posterior; Med, medial; Lat, lateral. False-color coding as shown on the color bars.

As we previously described in Fehérvári et al. (2015), a 50 μA current pulse at 250 μm depth (layer II/III) in V1 induced cortical activity around the stimulation site and in secondary, independent extrastriate spots surrounding the primary response. Cortical activity expanded around the response sites and widespread activation that encompassed all of V1 was observed. All V1 stimulation cases in this study showed this activation pattern, illustrated by one typical trial in Figures 1A–C. Figure 1A shows statistically significant (beyond 3 × SD of baseline noise) fluorescence activity following stimulation (50 μA at 250 μm depth, average of 12 repetitions) superimposed over the image of the cortical surface revealed through the cranial window. The primary response around the stimulation site in posterior V1 (relative to the visible cortical area) was surrounded by multiple spots of activity (arrows) on the lateral and medial sides. Response spots were labeled alphabetically from posterior to anterior, lateral sites first (A–G). Typically for V1 stimulation, cortical activity spread to cover most of the recorded region (frame at 50 ms after stimulation), and decreased thereafter (150 ms frame).

Fluorescence signal time courses were similar at all sites: a fast rising peak that gradually returned to baseline. This is essentially the same time course as described in our previous study (Fehérvári et al., 2015). Time courses at labeled sites in the example trial are shown in Figure 1B.

Independent cortical response sites were manually identified on latency maps at various thresholds between the level of significance (3 × SD) and 80% of peak maximum. Independent spots appeared as local latency minima on at least two latency maps ≥10% (of peak maximum) apart (see Section Latency Thresholds for Response Identification, Localization, and Measurement in Materials and Methods). For the example trial, Figure 1C shows the latency map of the imaged cortical region at 25% of peak maximum, with six independent spots (full arrows labeled A–G, except D). The protrusion on the contour line, pointed at by the hollow arrow with label D suggests an additional response spot, near which an independent response was present in other trials in the same animal. As response sites shifted slightly between different threshold levels, response locations were calculated on the 50%-latency map to preserve comparability between trials. The location of a response was defined on the latency map as the center of a response's independent area within the initial 1 ms of latency.

### Spatial relationship between V1, B/LM, and C/AL responses

Changes in the location of the V1 stimulation site caused changes in the location of the secondary response spots. In general, all secondary responses exhibited such shifts, and two sites, labeled B and C on Figure 1, had a clear enough pattern for associating them with known visual areas. These response sites' locations coincided with areas LM and AL on mouse visual area maps (Wang and Burkhalter, 2007; Polack and Contreras, 2012) and the observed shifts were in accordance with the visuotopic maps of the involved areas (V1, LM, and AL). To emphasize this, we refer to these two sites as B/LM and C/AL. As known, the visuotopic map of LM is mirrored to that of V1 along the shared lateral V1-LM border, and the visuotopic map of AL is inverted along both V1-AL and LM-AL borders (Wang and Burkhalter, 2007). Therefore, a shift in V1 from posterior to anterior is expected to be accompanied by an anterior shift in B/LM and a posterior shift in C/AL, moving them closer to each other. A medial-to-lateral shift in V1 is expected to be followed by an inverted, lateral-to-medial shift in both B/LM and C/AL, and the same is true for the opposite directions. No two other lateral areas have this mapping. This is a simplification of the more complex visuotopic maps of V1, LM, and AL, however, the described shifts sufficiently identify these areas with a limited number of stimulated locations in V1.

**Figure 2:**
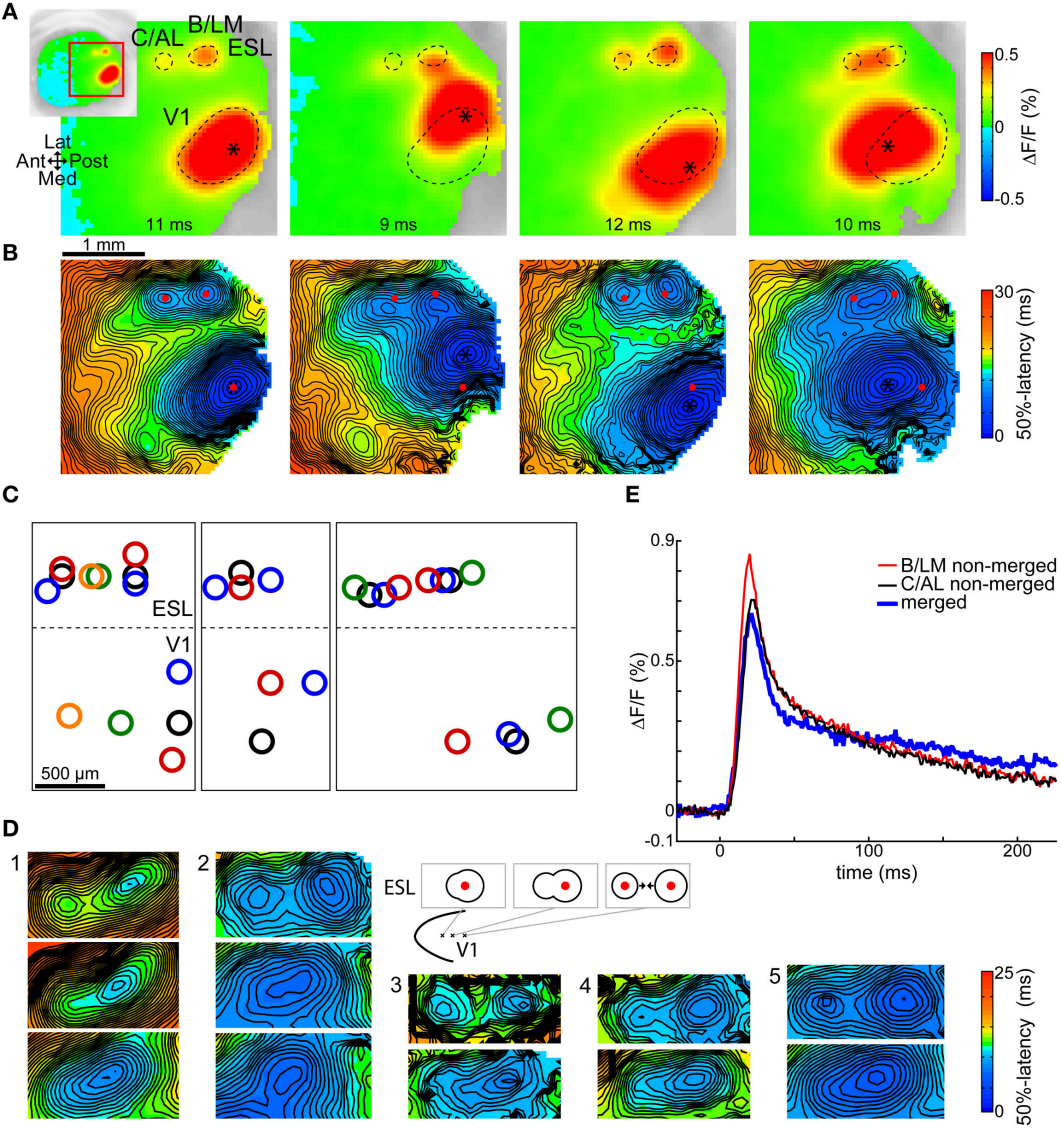
**Shifts of B/LM and C/AL responses following changes in stimulation site location**. Shifts in **(B,C)** locations were in accordance with known visuotopic maps of LM (inverted relative to V1 map along V1-LM border) and AL (inverted along V1-AL and LM-AL borders relative to V1 map). **(A)** VSD signal images taken at shown delays after stimulation, and **(B)** respective 50%-latency maps in the ROI (indicated by inset) in four trials in the same animal, each with a different V1 stimulation site location (asterisk). Contours (**A**, dashed curves) and locations (**B**, red dots) of the responses in the leftmost frames are overlaid on all other frames. Data from a different mouse than in Figure 1. **(C)** Each sub-panel: response locations from multiple trials in the same animal; data from three mice. Same-color rings belong to the same trial. Dashed lateral V1 borders are visual guides only. See Supplementary Figure 1 for additional data. **(D)** 50%-latency maps showing B/LM and C/AL response shifts, limited to the ROI, in five mice. Top rows show separate responses, lower rows show how these merged as the stimulation site was moved (further) in anterior or anterolateral direction (as indicated on the schematic). The responses are expected to shift in opposite direction (to each other) as the visuotopic maps of LM and AL are inverted at their common border. False-color coding as shown on the included color bars. **(E)** Comparison of merged and not merged B/LM and C/AL response time courses. Shown merged and not merged data are from two different trials in the same mouse. The merged and not merged time courses are very similar; the amplitude differences can be explained with variation between trials. ESL, ESM, lateral and medial extrastriate region, respectively.

Figure 2A shows the observed shifts in B/LM and C/AL in four trials from one animal, each with a different stimulation site in V1. Relative to the situation on the leftmost image, V1 stimulation sites (asterisks) were shifted to lateral, medial, and anterior directions, in this order from left to right. The outlines of the V1 primary, and B/LM and C/AL secondary response spots on the first image were copied to the other images for reference. Figure 2B shows 50%-latency maps of the same trials. Here, red markers indicate the centers of the V1, B/LM, and C/AL responses on the first image. Other sites present on these recordings were left unmarked. It can be seen on both VSD images (Figure 2A) and latency maps (Figure 2B) that when the V1 stimulation site was shifted laterally, both B/LM and C/AL responses moved in the medial direction, whereas a medial shift of the stimulation site caused a slight lateral shift in these two sites. When the V1 stimulation site was moved anterior, the two responses merged into a single, elongated response between the original positions of the B/LM and C/AL sites. Figure 2C shows V1 and corresponding B/LM and C/AL site locations in multiple trials in seven mice. With some variation, possibly due to the variability of the layout of mouse visual areas (Garrett et al., 2014) and to the inaccurate nature of stimulation, the above described expected shifts can be observed in each. This pattern could be demonstrated in all mice where enough data was available (≥3 stimulation locations in a cross pattern, or multiple sites along one axis; in total 10 mice, of which three are presented here; see Supplementary Figure 1 for data from all 10 mice).

Figure 2D illustrates the merging process with several examples. In each example each row corresponds to different trials in the same animal, and displays a section of the 50%-latency map limited to the region of interest (ROI). The top row shows separate B/LM and C/AL responses. In the second row, the V1 stimulation site was moved in anterior or anterolateral direction, which caused a single response to appear. The area of shortest latency of these single responses was typically closer to the posterior end, which is expected as in most cases B/LM had a shorter latency than C/AL (see Section Activation Order of Extrastriate Responses). In each further row, where present (examples 1 and 2), the stimulation site was moved further in the same direction, which shortened the anteroposterior extent of the single response. The most likely explanation of this process is two responses moving closer to each other.

Visual areas in the mouse have a relatively small size and share visuotopic locations on opposite sides of common borders. As demonstrated for B/LM and C/AL, depending on the retinotopic location of the V1 stimulation, two responses in neighboring areas can be located near enough to be indistinguishable with our imaging technique. A stronger response could hide a nearby located weaker one. In a similar fashion, response peaks elicited by visual stimulation stretched over visual area borders (Polack and Contreras, 2012). The relationship between observed responses and visual areas is therefore not clear, and some responses could have originated in multiple visual areas.

Our analysis, however, was exclusive to the earliest activating, central part of activated areas. In case of B/LM and C/AL, there are several factors that strongly indicate that their central responses originated in one visual area only and were not substantially affected by other areas: (1) the time course of merged responses was very similar to that of not merged ones. An example of this from two trials in the same mouse is shown in Figure 2E. Here, amplitude differences between merged and not merged cases can be explained by variation between trials, otherwise the time courses are very similar, and no summation effect is visible. Overall, peak shape features in merged and not merged (B/LM and C/AL) cases were not significantly different (time to peak, time to decay to half-peak, peak maximum ΔF/F, peak width at 50%; pairwise two-sample *t*-tests, *P* < 0.005, *n* = 60 for merged and *n* = 44 for B/LM and C/AL each). (2) B/LM and C/AL exhibited response shifts as expected from their retinotopy. (3) We never observed a separate response lateral from B/LM where area LI is expected, even at medial positions of B/LM. It is possible that the LI response was always masked by the stronger B/LM, but in that case it was too weak to strongly affect the central response of B/LM. Naturally, when B/LM and C/AL responses merged, the origin of the merged response center was unclear, therefore we omitted merged cases from further analysis altogether.

Regarding other response sites, we cannot say with certainty that all originated in only one area, however, the lack of any observed merging between these responses, and that response clusters were relatively far from each other suggests that it was the more probable case.

### Topographic map of extrastriate responses

**Figure 3:**
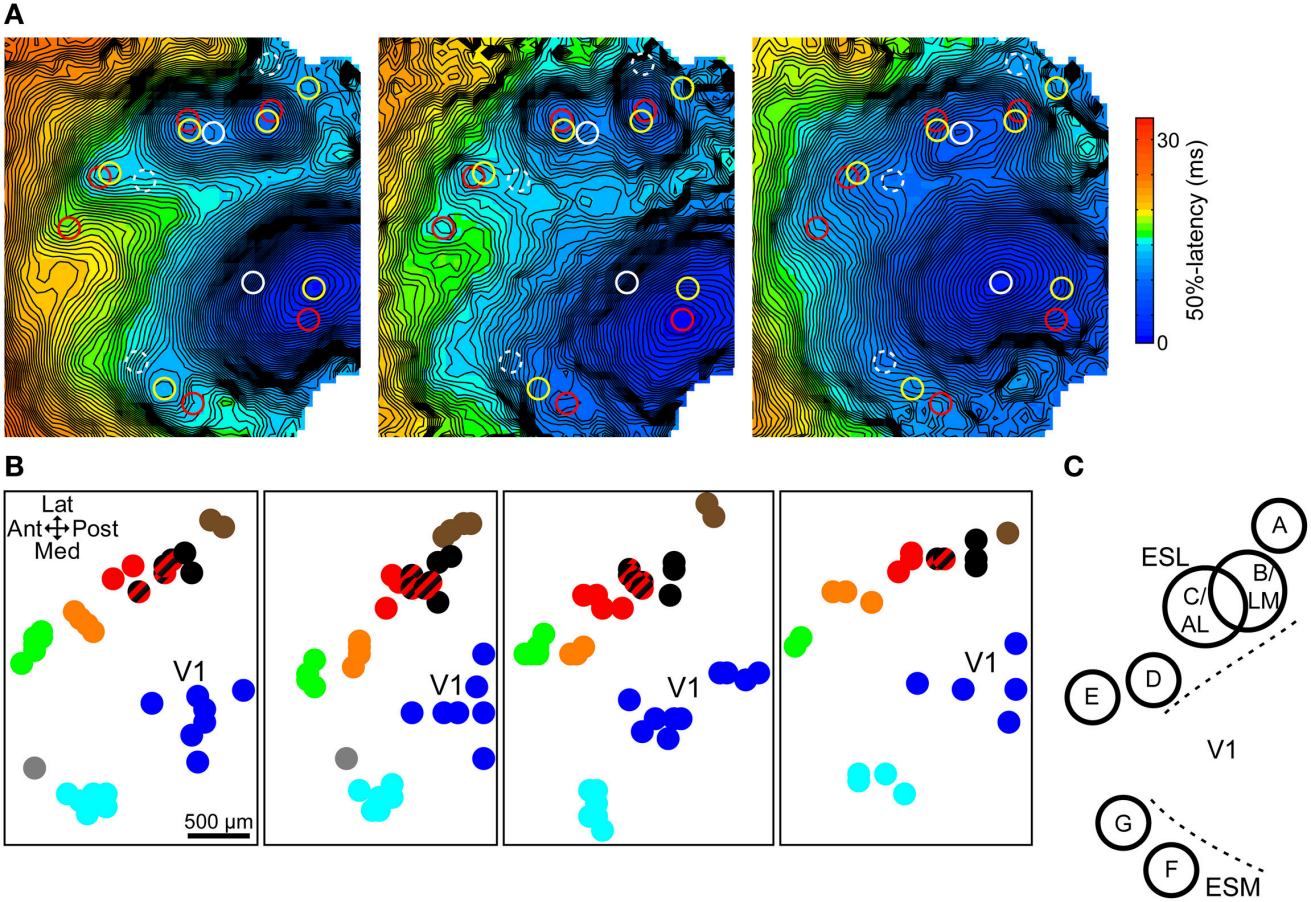
**Topographic map of extrastriate responses**. **(A)** 50%-latency maps of three trials in the same animal, each showing the response sites from all three, marked with circles. Same color circles are from the same trial. Possible but not independent sites are marked with dashed circles. Response cluster maps in **(B)** were created from marked locations like these. **(B)** Response site locations, determined on 50%-latency maps, in multiple trials in four mice. Response clusters were defined based on simultaneously present responses and proximity. Cluster members share the same color. Striped black/red spots indicate merged black (B/LM) and red responses (C/AL). **(C)** Approximate spatial pattern of observed extrastriate response clusters. Overlapping B and C circles indicate that merging of responses was only observed between these two sites. ESL, ESM, lateral and medial extrastriate region, respectively.

In order to identify the spatial pattern of the extrastriate secondary responses, response site locations were analyzed across all trials for each animal. Responses were grouped into clusters with the help of multiple responses simultaneously present in one trial, used to define clusters, and by proximity to already existing clusters. In addition, other parameters such as shape, latency, and size were also used to decide cluster membership. In general, clusters were located relatively far from each other and did not invade each other's area. Figure 3A demonstrates the process in three trials from the same animal. Response locations were marked on each trial's latency map and copied to the others for reference. Simultaneously present responses define clusters A–D and F in one trial (yellow circles) and B–F in another (red circles). Its elongated area between clusters B/LM and C/AL, latency, and the position of the stimulation site (anterior shift relative to the other sites, which causes B/LM and C/AL to move toward each other) helped identify the lateral independent response in the third trial (white circle) as a merged B/LM and C/AL response. Response clusters in four mice are shown in Figure 3B.

B/LM and C/AL responses, separate, or merged, were present in almost all trials (~98%, 104 out of 106 total trials) and were easily identifiable by the stereotaxic location and shifting pattern (see previous section). As such, these were used as a reference point relative to which other clusters could be identified across animals. Overall, we found five clusters laterally, and two medially from V1, comprising a pattern that was consistent in all animals (Figure 3C). As in earlier examples, labels A–G were assigned posterior to anterior, lateral first. Out of the total 33, sites A–G were present in 5, 32, 22, 19, 23, 32, and 5 animals, respectively. Additional response sites, visible in only 1–2 trials were not considered reliable enough to be included. The overlapping circles of clusters B/LM and C/AL in Figure 3C, and the mixed-color spots (black and red stripes) on Figure 3B indicate merged responses, which were only observed between these two sites. The location of the clusters resembles mouse anatomical visual area maps (Wang and Burkhalter, 2007) and the location of visually-evoked responses in a previous VSD imaging study (Polack and Contreras, 2012).

### Activation order of extrastriate responses

**Figure 4:**
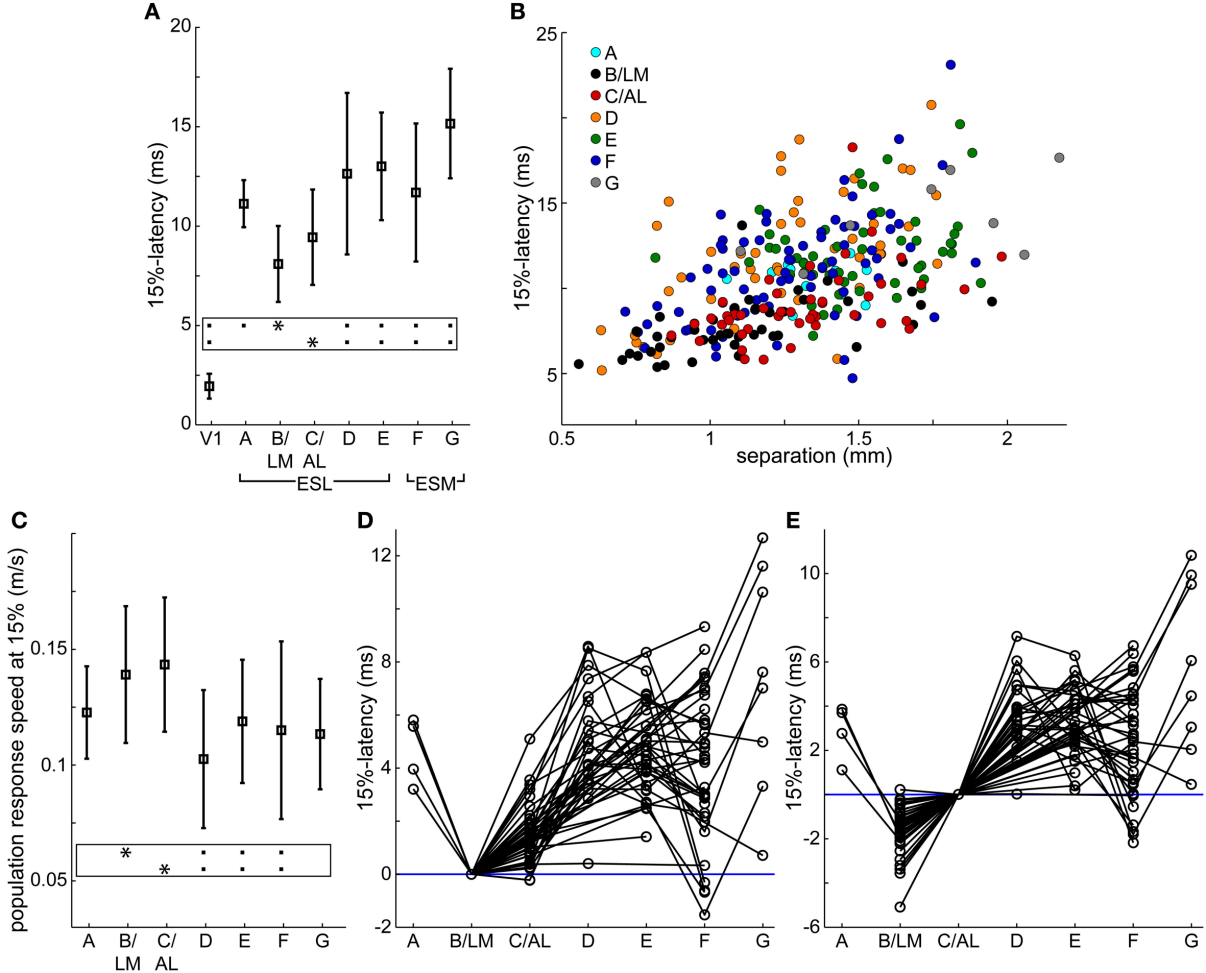
**Response latencies and population response transmission speeds. (A,C)** Average (square) ± SD (bars) latencies after stimulation **(A)** and population response transmission speeds **(C)** at V1 and extrastriate response sites at 15% of peak maximum in all 33 mice. Population response transmission speeds calculated as separation between stimulation site and response divided by latency. Same data as in Table 1. Frames: black squares in each line show statistically significant differences between the site marked by asterisk and other sites, e.g., in **(A)** site B/LM was significantly different from all other sites except C/AL. **(B)** 15%-latency vs. separation, data from all 106 trials. Colors indicate response area. **(D,E)** extrastriate response latencies relative to B/LM **(D)** and C/AL **(E)** in individual trials. Data points from the same trial are connected. Blue horizontal lines were added at 0 ms latency for visual guidance. In all panels, B/LM data were restricted to unmerged cases (C/AL also present, *n* = 44 trials), and only these trials are shown in panels **(D,E)**.

In recorded VSD videos, evoked cortical activity typically appeared first laterally from V1, near site B/LM (separate or merged), and appeared to spread in anterior (sites C/AL, D, and then E) and posterior (site A) directions. On the medial side, site F typically became active after B/LM and C/AL, and activity appeared to spread toward anterior (site G). Latencies measured at 15% of peak maximum at response sites in all 33 mice, averaged over a 5 × 5 pixel^2^ area, reflect this order (Table 1 and Figure 4A). Indicated statistics for B/LM only include non-merged cases, where C/AL was also discernible. This threshold was chosen because it provided information about early activation and was still above the level of statistical significance (3 × SD of baseline noise) in most of our detected responses (see Section Latency Thresholds for Response Identification, Localization, and Measurement in Materials and Methods).

**Table 1:** **Response latencies and interareal population response transmission speeds following V1 stimulation**.

**Response site**	**V1**	**A**	**B/LM**	**C/AL**	**D**	**E**	**F**	**G**
*n* (trial)	106	13	44	44	49	54	75	8
15%-latency (ms)	1.9 ± 0.6	11.1 ± 1.2	8.1 ± 1.9	9.4 ± 2.4	12.6 ± 4	13 ± 2.7	11.7 ± 3.5	15.2 ± 2.8
Population speed at 15% (m/s)	n/a	0.123 ± 0.02	0.14 ± 0.03	0.143 ± 0.029	0.103 ± 0.03	0.119 ± 0.027	0.115 ± 0.038	0.113 ± 0.024
Distance from V1 (mm; mean and range)	n/a	1.39, 1.06–1.75	1.12, 0.56–1.95	1.33, 0.87–1.98	1.23, 0.63–1.76	1.5, 0.82–1.91	1.27, 0.71–1.89	1.7, 1.1–2.18
*n* for distance from B/LM (trial)	44	4	n/a	44	23	30	36	8
Distance from B/LM (mm; mean and range)	1.12, 0.56–1.95	0.61, 0.45–0.70	n/a	0.49, 0.22–0.78	0.99, 0.66–1.37	1.33, 1.05–1.78	1.98, 1.76–2.21	1.93, 1.69–2.34

Latencies, given as mean ± SD, were measured from stimulation trigger to 15% of the response peak maximum at each site (V1, and A–G). Population response transmission speeds (shortened as population speed) were calculated as distance between electrode and response site, divided by latency, in each trial. Each trial was the average of 10–24 repeated stimulations. Total number of trials was 106 in 33 mice. N-values for each site reflect the number of trials in which such responses were observed. Data for B/LM and C/AL only include the 44 trials in which both responses were present, possibly merged cases were excluded (60 trials, see Spatial Relationship between V1, B/LM and C/AL Responses). The n-values for distances from B/LM were different, as some sites were present in trials when B/LM was not, and vice versa.

Mean latencies from all mice indicate that site B/LM was the fastest to activate, and C/AL the second fastest. Statistical comparison of mean latencies showed that B/LM and C/AL were not significantly different, B/LM had significantly faster latencies than all other sites, and C/AL was significantly faster than sites D–G (ANOVA, followed by the Tukey-Kramer test, *P* < 0.05).

We also looked at the activation order in individual trials. Latencies were rounded to the nearest millisecond to add tolerance. At 15% threshold, B/LM activated first alone (i.e., before all other sites) in 35 (~80%), and first together with another site in 5 (~11%) trials (*n* = 44). C/AL was second alone in 32 (~73%) trials, second together with another site in 5 (~11%) trials (*n* = 44).

### Population response transmission speed

Secondary response latencies are plotted against distance between V1 stimulation site and response location (separation) in Figure 4B. Data points from sites B/LM and C/AL are mostly found near the lower-end of the latency spectrum at any distance, in accordance with the activation order described above. This plot also reveals an uneven distribution of data points of each site along the distance axis. For example, B/LM, C/AL and F data points have smaller separation in general than those of site E. The reason for this is that the majority of stimulation sites were located in the wider, and easier-to-target posterior part of V1. To eliminate the bias caused by this uneven distribution, transmission speeds of VSD population response (population response transmission speed) were calculated, defined as separation divided by latency. The term “population response” was chosen to emphasize that this statistic only reflects the speed at which the fluorescence signal appeared at secondary response sites, which is not necessarily the same as, for example, axonal conduction velocity, as it may be influenced by other factors (see Section Possible Interpretations of Population Response Transmission Speed in Discussion). Mean ± SD population response transmission speeds at 15% are listed in Table 1 and shown in Figure 4C. Mean transmission speeds to B/LM and C/AL were faster than to other sites. Statistical analysis of speed means did not show a clear picture: B/LM and C/AL were not significantly different from each other, and B/LM and C/AL were significantly faster than D, E, and F, but not A or G (ANOVA, followed by the Tukey-Kramer test, *P* < 0.05). In individual trial data, sites B/LM and C/AL had the highest speed in ~77% of the trials at 15% threshold (*n* = 44 trials; B/LM was highest in 21, C/AL in 13 trials).

Both latency and transmission speed data indicate that in general B/LM and C/AL had faster activation from V1 than other sites.

### Direct transmission between V1 and extrastriate sites

We compared the possibility of direct vs. indirect transmission of the VSD signal to secondary responses. By indirect transmission we mean that the VSD signal passes through an additional, extrastriate relay site as opposed to direct V1-extrastriate transmission.

For relayed transmission through a V1 → relay → target path, the difference of V1 → relay and V1 → target latencies has to be long enough to allow for transmission between relay and target. We estimate the minimum necessary time for this to be around 3–4 ms (see Section Direct Input from V1 to Extrastriate Areas in the Discussion for explanation of this limit and further considerations on this topic). Latency differences in individual trials at 15% between B/LM and other sites, and C/AL and other sites are shown in Figures 4D,E, respectively. These latency differences indicate the time left for relay → target transmission if the relay site is B/LM or C/AL.

For B/LM, data points below the required minimum time at C/AL, E, F and G suggest direct transmission as there is not enough time from B/LM to transmit to these sites. Data points with larger latency differences could be relayed through B/LM. At sites A and D, there are data points around the 3–4 ms limit, but the decision is not clear. For C/AL, the latency differences are smaller, and data points below the limit suggest direct transmission at all sites. Again, higher latency differences could be relayed through C/AL. Other sites were not considered as relay sites because their longer latencies made that unfeasible.

### TTX in B/LM

**Figure 5:**
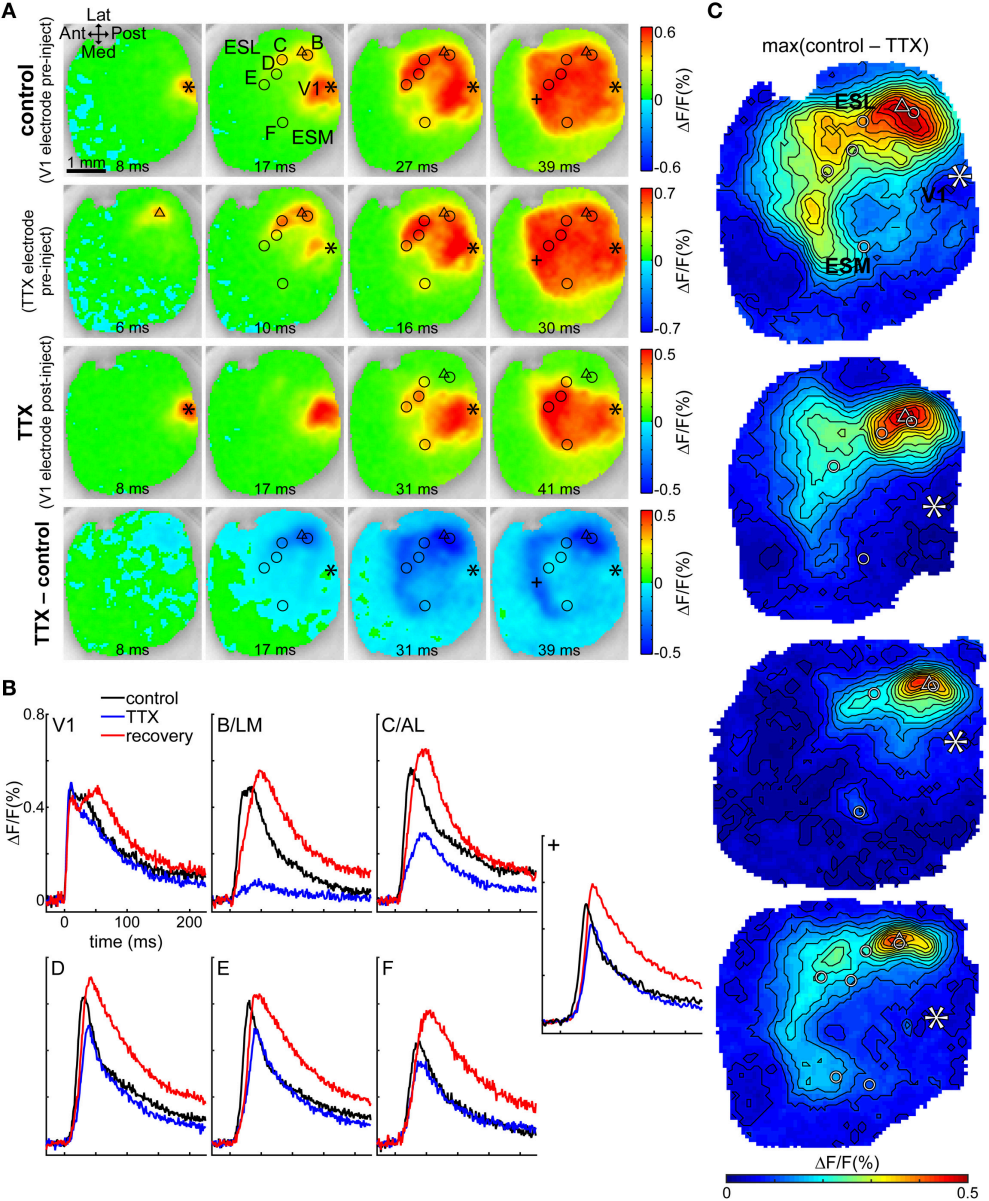
**TTX in B/LM reduces response amplitude in extrastriate areas**. **(A)** VSD images of evoked cortical activity at indicated delays after stimulation; each row shows a different trial in the same animal. First row (*control*), V1 electrode stimulation before application of TTX; second row, TTX electrode stimulation before application of TTX; third row (*TTX*), V1 electrode stimulation after application of TTX; fourth row (*TTX–control*), difference of V1 electrode stimulations pre- and post-application of TTX. Note that the color scales of the rows and the delays of frames in columns are slightly different. **(B)** Time courses of the fluorescence signal in V1 and labeled extrastriate sites. Data in **(A,B)** are from the same mouse. **(A,B)** Location “+” is an additional site to show the origin of the band appearing anterior to the visual cortex. **(C)** Maps of the maximum difference between V1 control and TTX trials in all four mice, demonstrating the spatial distribution of the strength of the TTX effect. **(A,C)** Asterisk, V1 stimulation site; triangle, TTX electrode for injection and stimulation; circles, extrastriate response sites as observed after V1 stimulation; Ant, anterior; Post, posterior; Med, medial; Lat, lateral; ESL, ESM, lateral and medial extrastriate region, respectively. False-color coding as indicated on color bars.

To further investigate direct vs. indirect transmission through B/LM, we injected TTX into layer II/III at depth 250 μm at the B/LM response site in four mice and observed how this affected the cortical activity evoked by stimulation in V1. This process is illustrated in one animal on Figures 5A,B (also see Supplementary Video 1). First, the stimulation electrode was inserted into V1 at a location where non-merged B/LM responses were induced. Then, the electrode containing ACSF + TTX (under a retaining current) was inserted at B/LM at 250 μm depth. For targeting, brief trials (2–3 repetitions with the TTX electrode, same stimulation parameters as on V1 electrode) were recorded to gauge the location of the electrode tip. Recordings of stimulation with the V1 and TTX electrodes at their final positions and before TTX ejection are shown in Figure 5A (top row: V1 electrode stimulation at the asterisk mark, labeled *control*; second row: TTX electrode stimulation at the triangle mark). After this point neither electrode was moved. Stimulation with the TTX electrode near B/LM induced cortical activity at extrastriate response sites and in V1, and the activity also spread to a large area, similarly to the pattern seen after V1 stimulation. Following this, TTX was delivered iontophoretically by the application of an ejecting current on the TTX electrode (see Section Tetrodotoxin Electrophoresis in Materials and Methods). Cortical response to V1 stimulation after the injection of TTX can be seen on the third row (labeled *TTX*). Here, fluorescence changes near the TTX electrode (triangle) were largely eliminated in recorded layers (see Discussion Activity in Deep Cortical Layers and Extent of TTX Effect on possible hidden activity in deeper layers). The bottom row visualizes the effect of TTX through the difference of the pre- and post-injection V1 stimulation data (*TTX–control*). The strongest effect was observed in this case around the injection site, then progressing on to the extrastriate area while V1 was less effected. The dark blue stripe anterior from the visual cortex on the 39 ms frame is due to a delay between *control* and *TTX*, as illustrated by the time courses at location “+” (Figure 5B, also indicated on Figure 5A).

Time courses at V1 and secondary response sites in the same animal show that TTX reduced the amplitude of the evoked fluorescence peak at secondary response sites (Figure 5B; locations are shown with circles in Figure 5A). Quantitatively, for all four mice, amplitude reductions at each site were (% of the original ± SD; *n*-values indicate number of mice the sites were observed in): B/LM was reduced to 16.9 ± 3% of original (*n* = 4); C/AL: 47.4 ± 18.9% (*n* = 4); D: 59.3 ± 8.2% (*n* = 2); E: 55.3 ± 6.3% (*n* = 3); F: 68.6 ± 15.5% (*n* = 4); G: 58.1% (*n* = 1); V1: 107 ± 8.7% (*n* = 4). The ratio of amplitudes before and after TTX application was tested against a zero mean (after/before—1; one-sample *t*-test, *p* < 0.05), and the reduction at sites B/LM (*n* = 4, *P* < 0.01), C/AL (*n* = 4, *P* = 0.01), E (*n* = 3, *P* < 0.01), and F (*n* = 4, *P* = 0.03) were significant; V1 (*n* = 4, *P* = 0.2) and D (*n* = 2, *P* = 0.09) were not significantly affected. G (*n* = 1) could not be tested. Peak delays were not significantly affected (two-sample *t*-test, *P* < 0.05).

The difference images (Figure 5A, fourth row) suggest that the TTX effect was concentrated laterally around the injection site, but medial sites and V1 were also somewhat affected (see 31 ms frame). Maps of the maximum effect (maximum of the difference between the V1 control and TTX recordings) in all four mice show (Figure 5C) the largest difference in lateral, anterior and anteromedial extrastriate regions, whereas V1 was much less affected. Interestingly, in 3 of 4 mice, response medial site F was also not strongly affected (as also indicated by a smaller quantitative reduction above). The uneven distribution of the reduction effect cannot be explained by simple diffusion of the TTX from the injection site. It is more likely that the secondary sites receive mixed inputs directly from V1 and B/LM, and TTX suppressed the latter.

Besides LM, AL is regarded as a possible gateway area to the visual processing streams in the mouse. For C/AL, relative latencies provided adequate proof of direct transmission from V1.

## Discussion

We used intracortical electrical stimulation in V1 layer II/III with VSD imaging in anesthetized adult mice to analyze transmission of cortical activity to extrastriate areas.

### Visual areas corresponding to response sites

The electrical stimulus induced cortical activity in V1 and in several extrastriate locations, in a pattern similar to known visual areas seen in anatomical and functional studies. We found five lateral-anterolateral extrastriate response sites (A–E) and two medial-anteromedial ones (F and G). Sites B and C were identified as visual areas LM and AL, respectively, by matching changes in their response location following changes in the V1 stimulation location to known retinotopic maps. For our conclusions, it was not required to accurately match other response sites to known visual areas. However, based on their relative location to V1, B/LM, and C/AL, and their shift patterns we surmise that D likely corresponded to area RL, E to A, F to PM, G to AM, and A was P or POR. We did not observe an independent response lateral from B/LM, where area LI was expected.

### Comparison with relevant studies

We compared our results to visually evoked responses also obtained with VSD imaging reported in Polack and Contreras (2012). Although intracortical electrical stimulation inarguably does not induce the same physiological response as visual signals, there were important similarities between the responses evoked with these different stimulation paradigms: locations of response peaks on the amplitude and latency peak maps presented in their study showed a remarkable resemblance to our response clusters, and activation order of extrastriate cortical regions also followed the same general pattern of ESL (analogous to our sites B/LM and C/AL) first, followed by ESM (analogous to F and G) and ESA (analogous to D and E). Based on their report and our own visual stimulation data, major relevant differences were: (1) activated area size limited in visual stimulation—our 50 μA stimulus was strong enough to induce a sustained, locally spreading activation wave not usually present at smaller intensities (Fehérvári et al., 2015); (2) longer interareal activation times, e.g., the visually evoked signal had a 17 ms latency difference between their central V1 response and ESL peaks—one reason for this is that the processing of visual signals follows complex cortical pathways compared to our stimulation paradigm in which projections from V1 to ES areas were likely directly stimulated; (3) response amplitudes decreased toward higher visual areas (largest in V1, smaller in ESL, smallest in ESA and ESM)—in our data there was no consistent difference between peak amplitudes (Figure 1B is not representative in this sense).

Transcranial electrical stimulation combined with endogenic flavoprotein fluorescence imaging (Hishida et al., 2011, 2014), and channelrhodopsin-2 photostimulation combined with VSD imaging (Lim et al., 2012) have revealed reciprocal transmission between V1 and higher visual regions. Their technique, however, was not able to discern individual visual areas within generic V2 regions (e.g., lateral V2, medial V2) or analyze latency differences between such areas, likely due to a relatively low time resolution (1 s after stimulation in case of flavoprotein imaging, 6.67 ms in the photostimulation study) or low spatial resolution in the ROI. Our study was able to observe and characterize responses in multiple individual visual areas within those generic regions using intracortical stimulation, which is a novel achievement.

It has been reported in ferret (Roland et al., 2006) and rat (Xu et al., 2007) that interaction between visual areas can also appear as a traveling wave that either traveled over area borders (Roland et al., 2006) or reflected from the V1/V2 border (Xu et al., 2007). Lateral spreading of cortical activity was observed around the V1 stimulation site as well as extrastriate responses, however extrastriate responses appeared before being reached by the lateral spreading from the V1 site, and after the lateral spreading typically engaged the whole of V1 and extrastriate areas, no secondary wave was seen. As we have also reported in an earlier study (Fehérvári et al., 2015) we were not able to observe waves traveling over or being reflected from the V1–extrastriate border in either direction.

### Possible interpretations of population response transmission speed

In this study, transmission from V1 to B/LM and C/AL responses was faster than to other response sites in terms of both latency and population response transmission speeds. Latencies, and through them transmission speeds, are measured by finding the delay at which the VSD fluorescence signal levels reach a chosen threshold. The VSD signal mainly reflects net subthreshold membrane potential changes in a population of neurons (Contreras and Llinás, 2001; Petersen et al., 2003). Therefore, measured latency can be decreased (and consequently transmission speed can be increased) by any factor which hastens the rise of local excitatory PSP levels—by increasing the number of activated neurons, causing earlier activation, reducing the amount of inhibitory PSPs, or by other means. Possible factors include e.g., faster conduction velocity, more targeted neurons due to a higher number of connecting fibers or more branching projections, lower number of inhibitory interneurons activated in the target area. Investigation of the exact reasons was not within the scope of this paper. However, areas AL and LM receive the strongest inputs from V1 (Coogan and Burkhalter, 1993; Wang and Burkhalter, 2007), which may be an important factor in the observed lower latencies and higher transmission speeds.

### Time courses show no difference between extrastriate areas

As the example in Figure 1B illustrates, evoked fluorescence changes in all extrastriate response sites had a qualitatively very similar time course, consisting of an initial peak and slow return to baseline. Apart from the reported latencies, our current data was not able to reveal characteristic differences between extrastriate areas, such as the ones reported in anatomical and functional studies (Andermann et al., 2011; Marshel et al., 2011; Wang et al., 2011, 2012; Wang and Burkhalter, 2013).

### Additional latency thresholds

Population response transmission speed calculations were also performed at various thresholds other than 15% of peak maximum: from 5 to 50% of peak maximum, at several absolute ΔF/F thresholds and at the level of significance (3 × SD of baseline noise). All of these calculations resulted in the same conclusions as reported in the Results Section. VSD imaging does not offer the same sensitivity and time resolution as more direct electrophysiological recording methods (e.g., local field potential, intracellular, and whole-cell recording). In accordance, our measured interareal transmission speeds (at the level of statistical significance, 0.21–0.22 m/s at B/LM and C/AL) were lower than the previously reported ~0.3–0.4 m/s in rat and mouse (Shao and Burkhalter, 1996; Nowak et al., 1997; Dong et al., 2004).

### Activity in deep cortical layers

The VSD signal mostly reflects cortical activity in layer 2/3 neurons as the dye does not penetrate to deep layers (Ferezou et al., 2006). Therefore, our data may have missed activity in deep layers. However, it has been reported that layer 2/3 stimulation in V1 induces mostly supragranular activity in LM in rat (Nowak et al., 1997) and mouse (De Pasquale and Sherman, 2011), it can be therefore assumed that our data covered the majority of the induced cortical activity. It is also likely that activity in deep layers propagates to superficial layers and affect the measured signal. The investigation of cortical activity in deep layers remains a subject for future study, which employs a different recording technique that penetrates deeper than the current VSD imaging.

### Extent of TTX effect

TTX injection largely eliminated the visible cortical response around the injection site (Figures 5A–C). On Figure 5C it can also be seen that the region of strongest effect (yellow to red shades) around the injection site was elongated along the anteroposterior axis (or posteromedial-anterolateral on the top image) and reached as far as C/AL in 2–3 cases, which raises the question whether the diffusion may have affected C/AL directly? The exact extent of diffusion is unclear, however, we believe it is unfeasible to assume that TTX diffused preferentially in the direction of elongation and not toward V1, for instance. The curvature of the cortical surface cannot account for such degree of elongation (Fehérvári et al., 2015). A more likely explanation is that the range of diffusion was shorter (probably comparable to the width of the region, not the length), in which case C/AL was too far for the diffused TTX to have a strong effect. The reason for elongation may lie in the shape of the region targeted by projections from the affected area.

At the same time, it is viable to assume that the injected TTX diffused in every direction, including to deeper cortical layers. Although the VSD signal originated in superficial layers and as such it does not directly prove that cortical activity was eliminated or significantly reduced in deep layers, it is very likely all layers were affected due to diffusion. As discussed in chapter 4.6, layer 2/3 stimulation induces mostly supragranular response in target areas, therefore it can be assumed that the majority of the induced cortical activity at B/LM was greatly suppressed by TTX.

### Direct input from V1 to extrastriate areas

In the anatomical two-stream model of mouse visual areas, defined by their unique connectivity profiles, areas AL, PM, RL, AM, and A belong to the dorsal stream, areas LM, LI, P, and POR are in the ventral stream (Wang et al., 2012; Wang and Burkhalter, 2013), and AL and LM are regarded as gateways of their respective stream (Wang et al., 2011). However, separation between the two streams is not perfect, as there is a high degree of interconnectivity between visual areas: basically every visual area has connection to all others (Wang et al., 2012). Furthermore, there appears to be a discrepancy between anatomical and functional classification of areas LM and PM (Andermann et al., 2011; Marshel et al., 2011; Roth et al., 2012; Wang and Burkhalter, 2013).

To test whether in our stimulation paradigm the evoked cortical activity was transmitted from V1 to secondary response sites directly (V1 → target) or relayed through a secondary site (V1 → relay → target), as a first step we analyzed latency differences between secondary sites. These differences were calculated from the measured V1 → secondary site 15%-latencies (Table 1) as V1 → relay latency subtracted from V1 → target latency for each considered relay and target site, and they reflect the remaining time for additional transmission between relay and target. We estimated the minimum required time for transmission between two sites to be around 3–4 ms: as the VSD signal predominantly consists of postsynaptic potentials (PSP), at least 1 ms is necessary for one synapse. Additional time is required for axonal conduction and for the PSP to rise to detectable levels (1–2 ms, time for conduction depends on distance). Furthermore, it has been shown in LM that the input (from V1) and output (to AL) neuron populations are distinct (Berezovskii et al., 2011). Assuming this was the case in visual areas in general, we added 1 ms to account for at least one additional synapse, ignoring an unknown length of transmission time as this is a lower estimate. Studies on visual cortical slice have reported onset latencies in LM following V1 stimulation ~4 ms in rat measuring local field potentials (Nowak et al., 1997) and in mouse with whole-cell clamp (Dong et al., 2004). Adjusting for shorter distances (see distances in our case in Table 1; ≥1.5 mm separation in the former, ≥800 μm in the latter study) and adding an additional synapse in the relay site, our estimate of 3–4 ms is feasible.

We only considered B/LM and C/AL as possible relay sites, as longer latencies at other secondary response sites made such assumption unfeasible. In case of C/AL, direct transmission was very likely due to small latency differences. In case of B/LM, the latency differences were not small enough to be conclusive at sites A and D. To take site distances in account, we also calculated estimated B/LM → target transmission speeds by dividing the B/LM-target distance (Table 1) by the latency difference (V1 → target latency minus V1 → B/LM latency) in each trial. Such speeds estimate how fast the signal had to propagate from the B/LM relay to the target sites if only the latency difference amount of time was left. The mean of the B/LM → target speeds were, at sites A and C–G respectively, 0.134, 0.42, 0.34, 0.34, 0.22, and 0.58 m/s. With the exception of the speed at site A, all these speeds are above the mean + 3 × SD of the maximum transmission speed we measured at 15% (V1 → C/AL, 0.143 ± 0.029), which indicates that transmission to sites B–G is unlikely to be exclusively relayed. In addition, suppression of the B/LM response with TTX reduced but did not eliminate other extrastriate responses. This strongly suggests that other responses were affected both directly by V1 and by B/LM.

These results indicate that, at least partially, evoked activity was transmitted directly from V1 to extrastriate areas. We believe that the direct connections reported in anatomical studies form the basis of the direct transmission seen in our experiments.

Areas in the anatomically defined dorsal stream occupy the medial, anterior and anterolateral extrastriatum, therefore it is possible that most of our observed response sites (C/AL–G) belong to this stream. Anatomically, LM and posterolateral higher visual areas (site A is likely one of these) are considered part of the ventral stream. On the other hand, in functional studies LM was found to have characteristics which do not clearly assign it to one stream. It is intriguing that the injection of TTX into B/LM affected all other observed secondary sites, because this could indicate that such interareal connections may also be active during visual processing, which suggests that LM is connected to both streams.

### Antidromic propagation

Projections between V1 and higher visual areas are reciprocal. As a consequence, electrical stimulation is likely to have triggered both orthodromic and antidromic interareal transmission. Antidromically activated neurons can potentially contribute to the fluorescence signal directly by membrane potential changes on their soma and dendritic arbor, and by synaptic activation of other neurons innervated by their local axon collaterals. Although contamination of our data by antidromic propagation cannot be completely excluded, in a rat visual cortex slice study using electrical stimulation, antidromic activation was seen in only ~4% of recorded neurons even with strong (=2.5 T) stimulation (Shao and Burkhalter, 1996). This stimulation strength is similar to ours, as the 50 μA stimulus was roughly 2–5 times stronger than the minimum necessary for a detectable response. The authors also argued that, because of their much lower number, feedback (to V1) fibers were activated with a much lower probability. Considering this, we believe that the vast majority of our recorded VSD signals are from orthodromic activation, however further investigation is necessary to clarify this.

## Conclusions

In summary, we used intracortical electrical stimulation with VSD imaging in anesthetized adult mice to analyze interareal transmission in superficial cortical layers from V1 to higher visual areas *in vivo*. We found seven extrastriate response sites (five lateral, two medial) in a spatial pattern similar to area maps of the mouse visual cortex and, by shifting the location of V1 stimulation, demonstrated that the evoked responses in B/LM and C/AL were in accordance with the visuotopic mappings of these areas known from anatomy and *in vivo* studies. These two sites, considered to be gateways to their processing streams, had shorter latencies and faster transmission speeds than other extrastriate response sites. Our data also indicated that the VSD signal was, at least partially, transmitted directly from V1 to extrastriate sites. Our results provide *in vivo* characterization of the spatiotemporal dynamics of V1-extrastriate transmission in multiple higher visual areas.

## Author contributions

Experiments were designed by TY and TF. Experiments were performed by TF. Data was analyzed by TY and TF. Manuscript was written by TY and TF.

### Conflict of interest statement

The authors declare that the research was conducted in the absence of any commercial or financial relationships that could be construed as a potential conflict of interest.
