# Supplementary material for: Population Response Propagation to Extrastriate Areas Evoked by Intracortical Electrical Stimulation in V1
Source: Front Neural Circuits. 2016 Feb 12;10:6. doi: 10.3389/fncir.2016.00006 (PMC4751260; doi:10.3389/fncir.2016.00006)
Supplement: Supplementary file 2 [file DataSheet1.DOCX]

Supplementary Material

Population response propagation to extrastriate areas evoked by intracortical electrical stimulation in V1

Tamás Dávid Fehérvári^1^, Tetsuya Yagi^1*^

^1^ Bio-System and Device Laboratory, Division of Electrical, Electronic and Information Engineering, Graduate School of Engineering, Osaka University, Suita, Osaka, Japan

**^*^ Correspondence:** Tetsuya Yagi, Bio-System and Device Laboratory, Division of Electrical, Electronic and Information Engineering, Graduate School of Engineering, Osaka University, Suita, Osaka, Japan

yagi@eei.eng.osaka-u.ac.jp

# Supplementary Figures and Videos

## Supplementary Figures


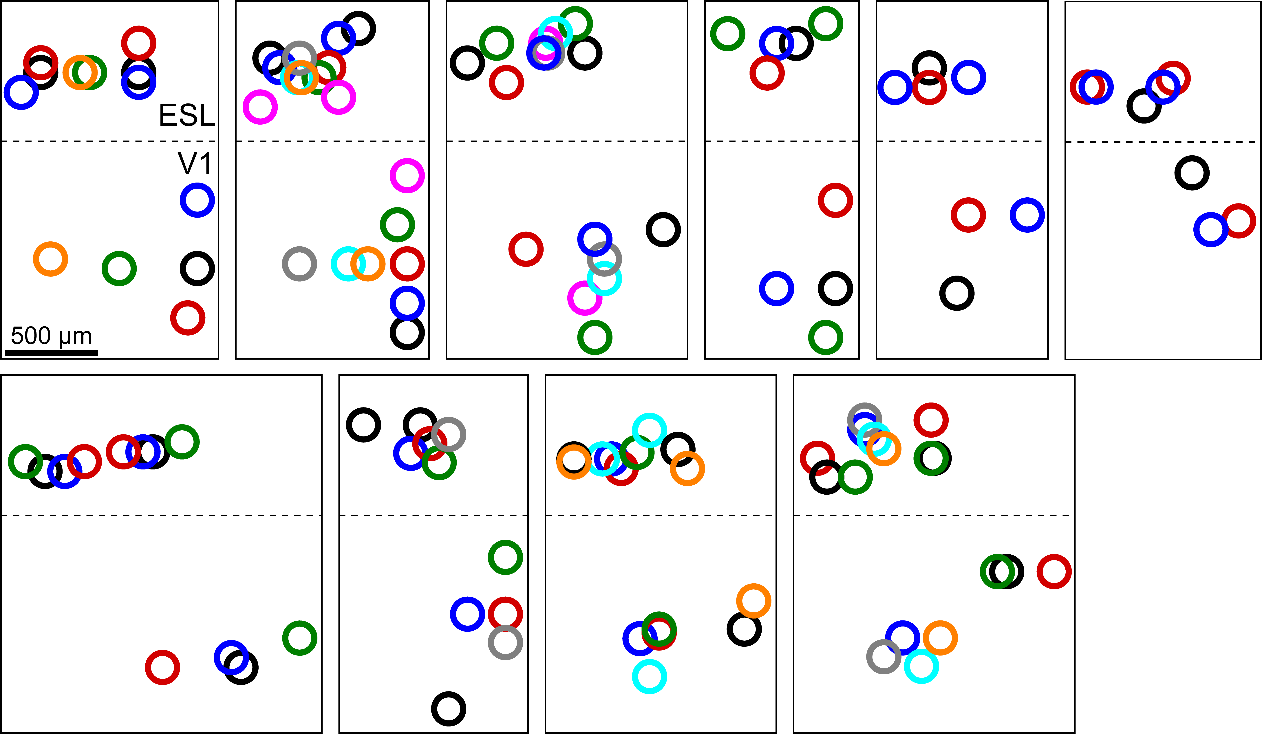


**Supplementary Figure 1.** **Shifts of B/LM and C/AL responses following changes in stimulation site location (additional data).** Additional data to Fig. 2C, showing shift patterns of B/LM and C/AL in all 10 mice where enough data was available. Each sub-panel shows multiple trials in one mouse; same colored rings belong to the same trial. The dashed lateral V1 border is for visual guidance only. ESL: lateral extrastriate region.

## Supplementary Videos

**Supplementary Video 1. Comparison of TTX trials.** Video of the recorded VSD signal in the four trials shown in Fig. 5A: V1 control, TTX control, TTX and V1 control-TTX difference. Color coding of each video is the same as that of the corresponding row in Fig. 5A.
